# Unveiling the Chemistry and Bioactivity of Bee Products and Their Derivatives

**DOI:** 10.3390/foods14173058

**Published:** 2025-08-29

**Authors:** Ofélia Anjos, Maria da Graça Miguel

**Affiliations:** 1 CERNAS-IPCB, Research Centre for Natural Resources, Environment and Society, Polytechnic University of Castelo Branco, 6000-084 Castelo Branco, Portugal; ofelia@ipcb.pt; 2 Centro de Estudos Florestais (CEF), Laboratorio Associado TERRA, Instituto Superior de Agronomia, Universidade de Lisboa, 1349-017 Lisboa, Portugal; 3 Centro de Biotecnologia de Plantas da Beira Interior, 6000-084 Castelo Branco, Portugal; 4 MED—Mediterranean Institute for Agriculture, Environment and Development & CHANGE-Global Change and Sustainability Institute, FCT, Universidade do Algarve, Edf. 8, Campus de Gambelas, 8005-139 Faro, Portugal

## 1. Introduction

Apiculture, or beekeeping, refers to the cultivation and management of honey bees for honey and byproducts, including the extraction, bottling, and sale of hive products such as honey, propolis, royal jelly, bee venom, bee pollen, bee bread and other fermented bee products [1].

Honey is a supersaturated solution or semi-solid natural sweet product produced by both honey bees (*Apis* subfamily) and stingless bees (*Meliponinae* subfamily) from carbohydrate-containing exudates produced by plants, mainly from nectar sources. The most common honey bee is *Apis mellifera* L., whereas stingless bees comprise multiple genera, including *Scaptotrigona*, *Melipona* and *Trigona*, with the last two being the most domesticated worldwide [1,2,3]. Honeydew honey is also a sweet natural product produced by bees from honeydew (sugary substance that aphids release on the bark or other parts of plants after assimilating the lymph) [1,2,3,4].

Honey is a complex mixture of carbohydrates along with other less common components like vitamins, minerals, lipids, organic acids, proteins, amino acids, flavonoids, pigments, waxes, pollen grains, various enzymes, and other phytochemicals. The first method for identifying the botanical source of bee honey is pollen analysis. With this information, one may identify pollen grains and use them to describe the honey-producing region [5].

According to the pollen analysis, honeys can be classified as (a) monofloral/unifloral if honeys contain predominantly pollen grains from an unique plant species (≥45% of all nectariferous pollen grains counted); (b) bifloral honeys contain pollen grains from two plant species with a frequency of 15–45% per nectariferous species; (c) plurifloral/multifloral honeys contain pollen grains from three or more nectariferous plant species with frequencies in the 3–15% (important minor pollen types,) or <3% (minor pollen types) [5]. Moreover, at the moment, ISO/TC 34/SC 19—Bee Products is working on developing an ISO standard for this purpose.

Melissopalynology’s time-consuming and tedious counting process makes it unsuitable for routine identification of honey origin. To guarantee the accuracy of the results, melissopalynology also needs highly skilled palynological specialists with in-depth experience and understanding of pollen morphology. Other approaches have been used to streamline the pollen identification process. The identification can be based on phytochemical compounds [carbohydrates, volatile compounds, phenolic compounds, organic acids, nitrogen compounds (amino acids and alkaloids), mineral elements] and biomacromolecular compounds (proteins, DNA), using chromatographic techniques and hyphenated techniques, spectral techniques, mass spectrometry, electrophoretic and immunologic techniques [6]. Other techniques include Fourier Transform–Near InfraRed (FT-NIR) spectroscopy on non-extracted samples; Fourier Transform InfraRed attenuated total reflectance (FTIR-ATR) spectrometer equipped with a deuterated triglycine sulfate (DTGS) detector on non-extracted samples; emission spectroscopy; and Nuclear Magnetic Resonance (NMR) spectroscopy on extracted samples [7]. Groups of homogenous samples are selected using statistical data processing in order to establish a standard profile for each type of honey sample under investigation, each of which has distinct organoleptic, microscopic, and chemical–physical characteristics. Comparing the data gathered on the honey sample to be evaluated with the previously determined profiles allows for the control of botanical origin because if the data overlap, the honey can be categorized as unifloral. Therefore, chemical analysis combined with chemometric processing enhances honey identification [7].

Honey is considered one of the most complex natural foodstuffs, and it is used as a food sweetener, complete food, nutraceutical agent, and medicinal supplement [1]. Although honey has been given a number of biological properties, including antimicrobial, antioxidant, anti-inflammatory, and immune-stimulating properties, and is also said to be useful in treating certain illnesses, such as arthritis, gastric ulcers, diarrhea, dermatitis, and wound healing, the evidence supporting its medicinal potential is complicated due to the action of various compounds and the notable variations in their amounts among honeys [8]. Manuka honey, which is made from the nectar of the *Lepstospermum scoparium* plant in New Zealand, is distinguished by having high concentrations of methylglyoxal (MGO) [9].

Apart from the compounds that comprise the honey, it also contains microorganisms. They can come from diverse primary sources such as microorganisms in the gut of bees, microorganisms collected from the environment they explore (soil, air, dust, plants, and flowers) to gather the compounds needed to produce honey (nectar, pollen, and plant sap), microorganisms inside the hive; and diverse secondary sources such as microorganisms that can contaminate honey while it is being extracted, handled by processing facilities, staff, and equipment, and storage [10]. Some examples of microorganisms that can appear in honey include *Actinetobacter* spp., *Bacillus* spp., *Clostridium* spp., *Corynebacterium* spp., *Pseudomonas* spp., the yeasts *Saccharomyces* and *Torula*, *Citrobacter* spp., *Enterobacter* spp., *Erwinia* spp., *Flavobacterium* spp., *Lactobacillus* spp., *Lactococcus* spp., *Leuconostoc* spp., *Listeria* spp., and *Pediococcus* spp. [11]. *Clostridium botulinum* spores, various *Bacillus* species, and, to a lesser extent, *Clostridium perfringens* spores have been documented among the sporulating bacteria in honey samples. *C. botulinum* can occasionally cause infant botulism in infants under a year of age because they are particularly susceptible to colonization by *C. botulinum* at an age between three to five months old [12]. *C. perfringens* can produce gas gangrene in deep wounds, which is a concern given the current usage of honey as a wound-healing agent [11]. Bees that fly into contaminated places, the use of contaminated equipment, or poor personal hygiene habits can also result in contamination with fecal bacteria [13].

Beyond the possible presence of pathogenic microorganisms, one of the main dangers of honey contamination is the existence of pesticide residues. As pollinators, bees may inadvertently collect nectar from plants exposed to pesticides, so adding those harmful compounds to the honey they produce. Honey may also contain antibiotics because beekeepers use them to treat diseases in bee colonies, which unintentionally contaminates the honey. Moreover, in densely populated and industrial regions, all bee products, including honey, may exhibit concentrations exceeding the acceptable limits for the majority of metals analyzed. Overall, honey producers and processors must possess a thorough understanding of honey contaminants and adhere to regulations that ensure the safety and authenticity of their honey products [14].

The complex chemical substance known as beeswax, which is produced by the glands of young worker bees aged 12 to 18 days, is used to form combs. In terms of chemistry, beeswax is a complex mixture of hydrocarbons, free fatty acids, free fatty alcohols, saturated and unsaturated linear and complex monoesters, and other minor elements. Approximately fifty separate fragrance components contribute to the unique perfume of beeswax, which also has a pleasant honey-like aroma [15,16,17]. Beewax can be used in cosmetics (lip balm, natural lip gloss), food processing (food preservative in the European Union, E 901), and apitherapy (coating of drugs and pills; mixed with drugs to retard drug release; chewing for strengthening the gingival tissue and to increase saliva and stomach juices) [18].

Propolis or bee glue is a sticky, resinous substance that bees use for construction and shelter. Propolis is a complex mixture of plant buds, resin and balsam (up to 70%), and other plant secretions and volatiles (<1% to 3%) that honey bees (*Apis* spp.) mix with pollen and beeswax (<10% to 87%) to create cerum, also known as cerumen. It can vary in color, texture, and odor from light to dark [19,20]. Native to tropical regions, stingless bee species (*Melipona* spp.) produce geopropolis, another kind of propolis, that combines soil components with beeswax and plant exudates [19]. The phytochemical composition of propolis varies significantly depending on geographical origin and plant species surrounding beehives. The primary sources of the propolis in temperate zones are plant bud resins from poplar species found in North America, Europe, non-tropical Asian countries, and New Zealand presenting similar phytochemical compositions, which are predominantly rich in flavonoids and phenolic acid esters, generally absent in those propolis samples from tropical regions [20,21]. According to the review made by Kasote et al. [20], in tropical regions, particularly South America, propolis samples are dominated by prenylated *p*-coumaric acid derivatives, flavonoids, benzophenones, lignans and terpenes. The comparatively varied chemical profile of samples from Mediterranean countries, such as Croatia, Algeria, the Island of Evia in Greece, and Cyprus, is characterized by high concentrations of diterpenoids and the absence or very low concentrations of flavonoids. Pacific propolis samples obtained from Taiwan and Japan (Okinawa) contain predominantly prenylated flavanones. Phenolic glycerides, specifically dicoumaroyl acetyl-, diferuloyl acetyl-, feruloyl coumaroyl acetyland, and caffeoyl coumaroyl acetyl glycerol, were found in propolis from North Russia and mountainous regions of Switzerland and Italy. All of these variations are due particularly to the vegetation that bees visit near their hives [20].

The volatiles present in propolis are more variable in their chemical composition, especially with respect to the relative quantities of different constituents [22]. In most European propolis samples studied, sesquiterpenes predominate in the volatile oils, such as β-eudesmol or viridiflorol from the south of Portugal, followed by aromatic compounds, such as benzyl acetate, benzyl benzoate and benzyl alcohol. However, the monoterpne α-pinene also occurs in propolis samples from Greece or Southern Italy (Adriatic coast), and Estonia. In this case, β-pinene and 1,8-cineole also occur in relatively high amounts. Labdane diterpenes are abundant in volatiles from some propolis samples collected in Portugal, mainly in *C. ladanifer*-rich regions [23,24]. In Morocco, β-eudesmol, cedrol, n-tricosane, and ar-curcumene could be detected in propolis samples [25]. In Chinese propolis, the major constituents of volatile oils are α-bisabolol, 2-methyl-3-buten-2-ol, and 3-methyl-2-butene-1-ol, 3-methyl-3-butene-1-ol and 3-methyl-2-butene-1-ol, 4-penten-1-yl acetate and the sesquiterpene α-longipinene. In propolis samples from Turkey, oxygenated hydrocarbons, oxygenated sesquiterpenes, aromatic alcohols and esters and the monoterpenes α-terpinene and α-terpineol have been reported. In Indian propolis, long-chain alkanes tricosane, hexacosane heptacosane heneicosane; the terpenoids linalool and geraniol; the phenylpropene methyleugenol; and phenols ((*Z*)-ethyl cinnamate) have been reported. The sesquiterpenes caryophyllene, spatulenol and δ-cadinene are the major compounds in the volatiles of several Brazilian samples [22]. In a review carried out by Miguel and Figueiredo [19], the authors provided an update on the volatiles that have been identified in both propolis and geopropolis from various geographic origins.

Many biological characteristics of propolis have been reported worldwide, such as its antimicrobial activity, which has led to its use in gynecological, dermatological, and oral diseases; its antioxidant qualities, which have led to its use in wound healing; and its immunomodulatory, anti-inflammatory, and antitumoral qualities, among others [1,26].

Royal jelly is a creamy-whitish fluid secreted by the hypopharyngeal and mandibular glands of young newly emerged workers honey bees (*A. mellifera*), typically 5–15 days old, which are called nurses [27]. Royal jelly is generally constituted by water (60–70%), proteins (9–18%), carbohydrates (7–18%), lipids (3–8%), minerals (0.8–3%), vitamins, phenols and amino acids. The changes in these metabolites within these ranges are attributable to numerous factors such as the heterogeneous nature of royal jelly, different sites where royal jelly is produced, and different times of production [28]. In royal jelly, it is possible to find major royal jelly proteins (MRJPs) that are water soluble, constituting more than 80% of royal proteins. Other proteins such as royalisin, and peptides (jelleins) are also constituents of royal jelly. Free amino acids are also found in royal jelly [28]. Fructose and glucose are the main carbohydrates of royal jelly (over 90% of the total sugars). In the lipid fraction, there are fatty acids such *trans*-10-hydroxy-2-decenoic acid (HDEA or 10-HDA) (32%), and 10-hydroxydecanoic acid (10-HDAA) (22%), along with sterols, with 24-methylenecholesterol as the most abundant. Volatiles can also be found in royal jelly, mainly including carboxylic acids, esters, aldehyde/ketones, and alcohols. For more detailed information, it is recommended to read the review on royal jelly’s volatiles [28].

Royal jelly has been used in medicine and cosmetics in addition as a functional food. Its use is continuously increasing [29,30]. It can be consumed as a dietary item or as a beneficial component of recipes. Various biological features, including antibacterial, antioxidant, immunomodulatory, aging delay, diabetes, obesity, hypertension, osteoporosis, and wound-healing activities, have been documented for royal jelly based on in vitro and in vivo experiments [29,30].

Bee venom is an odorless and transparent liquid containing a hydrolytic mixture of proteins with acid pH (4.5 to 5.5) that bees often use as a defense tool against predators. Bee venom is made by female worker bees and contains a variety of active compounds, including peptides like melittin, apamin, mast cell degranulating peptide, and adolapin, as well as enzymes like phospholipase A2 and hyaluronidase. Volatile compounds and amino acids are also present in bee venom [31]. The bee venom causes allergic reactions after the sting. The epidermis, respiratory tract, cardiovascular system, and gastrointestinal system may all experience these effects. Severe anaphylactic shock may then result in myocardial or cerebral ischemia. Many protein allergens (melittin, hyaluronidase, and phospholipase A2), the majority of which have enzymatic activity, cause allergic reactions. However, these compounds may also have therapeutic potential in the treatment of inflammatory disorders in humans, diseases of the central nervous system, including amyotrophic lateral sclerosis, Parkinson’s disease, and Alzheimer’s disease and anti-viral and anticancer properties against human immunodeficiency virus (HIV) and ovarian and prostate cancer [31].

Bee pollen is the pollen (male gametophytes generated by the anthers of flowering plants) that is moistened and aggregated into pellets that range in size from 1.4 to 4.0 mm by bees, one of the most prevalent pollinators, that combine the collected pollen with their own secretions and the nectar in plants [32]. Bee pollen contains macronutrients such as carbohydrates (24–60%), proteins (7–40%), lipids (1–18%) and fibers (0.3–20%), in addition to micronutrients such as essential amino acids, vitamins, minerals, fatty acids, phenolic acids, flavonoids, sterols, terpenoids and carotenoids. Although pollen is used by bees as a source of protein, it has recently drawn more interest as also a possible dietary source for humans. Therefore, bee pollen has been promoted as an alternative dietary supplement due to its high nutritional value. Nevertheless, bee pollen also possesses health-promoting properties, such as antioxidant, anti-inflammatory, antimicrobial, anti-carcinogenic, anti-atherosclerotic and hepatoprotective effects, due to the presence of phytochemicals like flavonoids, phenolic acids, and phenolamides [32,33,34]. The botanical origin, geographic location, and climate all have an impact on the composition of bee pollen, which in turn affects the bioactive qualities of this bee product [32].

Campos et al. [34] published a review on the standard methods for pollen research including the identification of the pollen’s floral sources, and determination of the more important quality criteria such as water content and content of proteins, carbohydrates, fatty acids, vitamins, alkaloids, phenolic and polyphenolic compounds. In addition, in the same article, the authors reviewed the methods for the determination of some bioactivities (antioxidant, anti-inflammatory, antimicrobial and antimutagenic properties) of bee pollen.

Bee bread is a mixture of pollen, honey, and bee salivary gland secretions sealed into honeycomb cells with wax and honey that undergoes lactic fermentation in the bee nest environment by lactic acid bacteria. Bee bread has a strong flavor and a caramel-like color. Due to its fermentation process, bee bread has a composition that is comparable to that of bee pollen but differs significantly in terms of amounts. For example, bee pollen is lower in vitamins, sugar, lactic acid, and amino acids than bee bread. Also, because the natural fermentation breaks down the multilayered wall of the pollen grain, bee bread is more physiologically active and easier for humans to digest and absorb [32,35,36]. Fermentation keeps bee pollen from losing its qualities and boosts the amount of healthy substances in the bee bread, which can be eaten or taken as a dietary supplement. The difficulty of extracting bee bread from the hive and the lack of natural bee bread production led to the development of artificially fermenting bee pollen [35].

According to the review by Durazzo et al. [1], mead and honey vinegar are fermented products obtained from honey. Mead is obtained via alcoholic fermentation using diluted honey and yeast and is recommended for people suffering from anemia and chronic gastrointestinal disorders due to its richness in nutrients and various components required by living organisms. Honey vinegar is made from mead (usually diluted) via acetic acid bacterial fermentation and presents characteristics similar to traditional wine vinegar.

*Abbamele* and *água-mel* are also bee-based products produced in Italy (Sardinia) and Portugal, respectively. After removing the honey, the honeycombs are broken and submerged in warm water (40 °C). The remaining liquid, which contains water, some honey, and pollen, is heated to 100 °C after the developing wax separates to obtain *abbamele*, a brown, honey-like substance that is sometimes flavored with the rinds of oranges or lemons [37]. Beekeepers in the southern area of Portugal make *água-mel* by first scalding the honeycomb with water while it still contains some honey, pollen, and propolis. After the honeycomb is removed, the honey/water solution is filtered through a sieve and simmered for a few hours, or until a dark brown honey-like product with the proper °Brix is achieved. This process is arbitrary and does not follow any industrial manufacturing guidelines [38].

## 2. Research Topic Overview

The Research Topic titled “Biological Activities and Chemical Composition of Bee Products and Derivatives” had six participating MDPI journals: *Antioxidants*, *BioChem*, *Current Issues in Molecular Biology*, *Foods*, *International Journal of Molecular Sciences*, and *Molecules*. Fourteen articles were published in this Research Topic.

These articles are presented below:

“Brazilian Stingless Bee Geopropolis Exhibit Antioxidant Properties and Anticancer Potential Against Hepatocellular Carcinoma Cells” by Muniz da Paz et al. [1] investigated the selectivity for tumor cells and anticancer efficacy of three geopropolis extracts from the meliponine bees *Melipona mondury*, *Melipona marginata*, and *Melipona bicolor* from Brazil in hepatocellular carcinoma (HCC) cells (Hep3B, HepG2, Huh-7, and PLC/PRF/5). This study was carried out because globally, HCC ranks sixth in incidence and third in number of deaths from cancer. The phytochemical analysis of geopropolis extracts analyzed using ultra-high-performance liquid chromatography coupled with high-resolution mass spectrometry (UPLC-QTOF HRMS/MS) revealed the predominance of the diterpenoids cupressic acid and 15-acetoxyisocuppressic acid, and the flavonoids isorhamnetin, dihydrokaempferide, and ferreirin. Propolis extracts of *Melipona bicolor*, *Melipona marginata*, and *Melipona mondury* demonstrated differential capacities to induce cytotoxicity to HCC cell lines in a two dimensional (2D)-cell culture model. This difference was related to the antioxidant activity found for these extracts. The best selectivity indices (SIs) were observed with Huh-7 cells, but geopropolis from *Melipona mondury* also showed a good SI on Hep3B cells. All geopropolis extracts exhibited anti-tumor activity presenting greater SI values than cisplatin, a drug used in HCC chemotherapy. To mimic tumor conditions, three-dimensional (3D) cell culture models were also employed. In these models, geopropolis extract from *Melipona mondury* was the only extract able to significantly reduce cell viability of Hep3B spheroids, as well as their size and outer layer morphology, compared to the control. In conclusion, geopropolis may be a good option for chemotherapy to treat HCC cells.

“Healing Activity of Propolis of Stingless Bee (*Scaptotrigona aff. postica*), Reared in Monoculture of Açaí (*Euterpe oleracea*), in Induced Wounds in Rats” by Ferreira et al. [2] investigated for the first time how propolis-based cream made from Amazon stingless bees, *Scaptotrigona aff. postica*, raised in acaí (*Euterpe oleracea*) monoculture, healed Wistar rats’ induced wounds. The treatment for seven days with the propolis-based cream when compared to that of collagenase did not differ significantly in terms of wound diameter reduction. In contrast to the positive control, the propolis-based cream had a less severe inflammatory reaction and had a direct impact on the lesion maturation process. This effect was possibly associated with antimicrobial and anti-inflammatory compounds identified by gas chromatography coupled to mass spectrometry (GC/MS) analysis in the propolis, such as the triterpenoids lup-20(29)-en-3-one (32.64%), lupeol (17.21%), 4,4,6a,6b,8a,11,11,14b-octamethyl-1,4,4a (13.16%); and lup-20(29)-en-3-ol, acetate, (3β)- (9.93%).

“Histological, Immunohistochemical and Antioxidant Analysis of Skin Wound Healing Influenced by the Topical Application of Brazilian Red Propolis” by Conceição et al. [3] investigated the healing action of the hydroalcoholic extract and paste, both at 1%, obtained from the Brazilian red propolis on skin wound excision model in male Wistar rats. Red propolis improved wound contraction, epithelialization, decreased crust formation, and altered the distribution of healing-associated components, mainly collagen I, collagen III, matrix metalloproteinase-9 (MMP-9), transforming growth factor beta-3 (TGF-β-3, and vascular endothelial growth factor (VEGF), according to macroscopical, histological, and immunohistochemical studies. The biochemical analysis showed that the Brazilian red propolis regulated the activity of the antioxidant enzymes superoxide dismutase, myeloperoxidase, and glutathione reductase, being similar to that of collagenase (positive control) but higher glutathione concentration than those treated with collagenase, therefore avoiding any oxidative stress that would prevent healing.

“Wound Healing, Anti-Inflammatory and Anti-Oxidant Activities, and Chemical Composition of Korean Propolis from Different Sources” by Dekebo et al. [4] investigated the chemical composition by GC-MS, and the antioxidant, wound healing, and anti-inflammatory effects of the ethanolic extract of Korean propolis collected in Andong (A), Gongju field (GF), and Gongju mountain (GM). With percentages higher than 5%, all extracts had as major components the flavonoids pinocembrin (12.0–17.7%), chrysin (5.2–6.8%), and apigenin (5.30–5.84%). According to the excision, incision, and dead space wound models using healthy adult Swiss albino mice of both sexes, the simple ointment with 10% GM exhibited the highest level of wound healing activity. When the inhibitory effect of nitric oxide (NO) production and anti-inflammatory activity were assessed, the GM sample gave the best results. With regard to the expression of inflammatory mediators such as inducible *nitric oxide synthase* (iNOS), interleukin-1 beta (IL-1β), and IL-6, GM and GF samples showed comparable effects. The presence of higher concentration of flavonoids in Korean ethanolic extract propolis might be responsible for their promising wound healing, anti-inflammatory, and antioxidant properties. In addition, the in vivo acute dermal toxicity studies on healthy adult Swiss albino mice treated with ethanolic extracts of propolis did not present any signs of toxicity, thereby granting approval for further clinical research.

“Propolis Ethanolic Extract Attenuates D-gal-induced C2C12 Cell Injury by Modulating Nrf2/HO-1 and p38/p53 Signaling Pathways” by Tian et al. [5] investigated the effect of propolis ethanolic extract on D-galactose (D-gal)-induced damage in the C2C12 mouse myoblast cell line. Through the activation of the Nrf2/HO-1 signaling pathway, propolis ethanolic extract prevented D-gal-induced oxidative stress and preserved the capacity of C2C12 cells to differentiate. By lowering p53 expression and inhibiting p38 phosphorylation, propolis ethanolic extract also prevented apoptosis. Additionally, propolis ethanolic extract increased the differentiation of C2C12 cells, reduced the quantity of senescence-associated β-galactosidase (SA-β-Gal)-positive cells, and increased the viability of senescent C2C12 cells.

“Inhibitory Effects of Aqueous Ethanol Extracts of Poplar-Type Propolis on Advanced Glycation End Products and Protein Oxidation” by Wang et al. [6] investigated the inhibitory effect of several aqueous ethanol extracts of poplar-type propolis on advanced glycation end products (AGEs), and oxidative modifications in bovine serum albumin (BSA)-glucose and BSA-methylglyoxal models. The findings showed that these propolis extracts were highly effective at preventing the production of pentosidine, Nε-carboxymethyllysine (CML), and total AGEs. Additionally, by assessing the amounts of carbonyl and thiol groups and examining tryptophan fluorescence quenching, the study found that these propolis extracts may successfully prevent oxidative modification, particularly propolis extracts in 75% ethanol that had the strongest inhibitory action, outperforming the inhibitor aminoguanidine. Investigating potent inhibitors is essential to stop protein glycation because one important factor in the development of AGEs and intermediates, which cause complications in diabetes, is the non-enzymatic glycation of proteins. Aqueous ethanol extracts of poplar-type propolis exhibited remarkable anti-glycation potency because of their high concentrations of phenolic compounds (benzyl caffeate, phenethyl caffeate, benzyl-*p*-coumarate, cinnamyl caffeate, and cinnamyl-*p*-coumarate), including flavonoids (pinobanksin, isorhamnetin, pinocembrin, pinobanksin-3-*O*-acetate, chrysin, and galangin), which scavenged free radicals, lowered reactive oxygen species (ROS), and scavenged reactive carbonyl species (RCS) during the protein glycation process.

“Chemical Composition of Volatile and Extractive Components of Canary (Tenerife) Propolis” by Isidorov et al. [7] investigated for the first time the chemical composition of propolis from Tenerife (Canary Islands) collected from different apiaries. The main groups of volatiles were formed by terpenoids, followed by C_6_–C_17_ alkanes, alkenes and aliphatic C_3_–C_11_ carbonyl compounds, C_1_–C_6_ aliphatic acids and C_2_–C_8_ alcohols, as well as their esters. Although terpenoids constitute the most important group in the volatiles of propolis, their content varied greatly amongst samples, most likely because the samples were taken from apiaries at varying altitudes. Propolis from Tenerife contained aromatic chemicals in the diethyl ether fraction obtained from the volatile fraction. Such compounds include alkyl- and alkenyl-substituted derivatives of salicylic acid and resorcinol, as well as a group of nine isomeric furofuranoid lignans. However, a nearly total absence of phenylcarboxylic acids and flavonoids was reported, which are typical of *Apis mellifera* propolis from various parts of North America and Eurasia. Such results may result from the vegetation of the Canary Islands, which is characterized by a large number of endemic species confined to different altitudinal levels.

“The Chemical Composition of *Scaptotrigona mexicana* Honey and Propolis Collected in Two Locations: Similarities and Differences” by Gerginova et al. [8] investigated the influence of flora available in the vicinity of the hives, the preferences of the stingless bee *Scaptotrigona nexicana*, and the climate (altitude and temperature) on the chemical composition and antioxidant activity of honey and propolis using nuclear magnetic resonance (NMR) and GC-MS, respectively. Samples from 24 colonies were analyzed: 12 each from two *S. mexicana* meliponaries located in the state of Chiapas in southern Mexico, approximately 8.5 km apart, Tuxtla Chico and Cacahoatán. The Tuxtla Chico honey samples contained higher concentrations of glucose and fructose, while the Cacahoatán samples displayed a rich composition of di- and trisaccharides. The usual *Mangifera indica* chemical markers were present in all samples from both locations, including the group of phenolic lipids, mainly cardols (alk(en)yl resorcinols), and cycloartane-type triterpenes (cycloartenol, mangiferolic, and somangiferolic acids). Nonetheless, there was also a notable distinction between the two sample groups. Eleven out of twelve samples from the Tuxtla Chico region had virtually no secondary metabolites other than compounds found in mango trees, whereas samples from the Cacahoatán region had small amounts of quinic acid isomers, dibenzylbutanediol lignans (dihydrocubebin and 3,4-methylenedioxy secoisolariciresinol), and kaurane type diterpenes (mainly kaurenoic acid). The results showed how geographic location, specifically plant origin and climate, affects the composition of propolis and honey produced by the same stingless bee species. The results also supported the assumption that bee species cannot be considered the most important factor in determining the chemical composition of propolis and honey.

“Antioxidant Activity, Physicochemical and Sensory Properties of Stingless Bee Honey from Australia” by dos Santos et al. [9] investigated the physicochemical and sensory attributes, total phenolic content, and antioxidant activity of 36 honey samples produced by two different stingless bee species (*Tetragonula carbonaria* and *Tetragonula hockingsi*) from Australia. The findings revealed moisture content across all samples ranges from 24.9% to 30.8% (*w*/*w*), electrical conductivity from 1.02 to 2.15 mS/cm, pH levels between 3.57 and 6.54, soluble solids from 69.2 to 75.1 °Brix, trehalulose concentrations from 6.20 to 38.2 g/100 g, fructose levels from 7.79 to 33.4 g/100 g, and glucose content from 3.36 to 26.8 g/100 g, and absence of sucrose. Trehalulose was present in every sample that was examined, which confirms the results of previous research that suggests this peculiar disaccharide as a honey hallmark from stingless bees. Additionally, research also showed that stingless bee honey contained phenolic compounds and antioxidant activity, highlighting the potential of these honeys as a natural antioxidant source. The study finds that the content of Australian stingless bee honey varies significantly depending on the time of harvest, geographic origin, and floral availability. Thirty participants in a sensory analysis thought that the sourness of Australian stingless bee honey was stronger than that of New Zealand Manuka honey.

“Network Pharmacology and Molecular Docking Analysis of Active Compounds in Tualang Honey against Atherosclerosis” by Azman et al. [10] investigated the potential pharmacological effects of the bioactive compounds catechin, ethyl oleate, fisetin, hesperetin, kaempferol, and luteolin on atherosclerosis, although a total of 103 candidate compounds have been identified in Tualang honey. The Swiss Target Prediction and SuperPred databases were used to search for the bioactive chemicals in Tualang honey and forecast their putative gene targets. GeneCards, DisGeNet, and OMIM databases were used to obtain atherosclerosis genes. Kyoto Encyclopedia of Genes and Genomes (KEGG) pathway analysis, gene ontology, and protein–protein interaction were used to determine how those compounds interacted with the genes responsible for atherosclerosis. Molecular docking investigations using the AutoDock Tools program were then used to further validate the findings of the analyses. The results demonstrated that, using network pharmacology analysis, 238 possible targets for the therapy of atherosclerosis with bioactive chemicals detected in Tualang honey were found. The network diagram also identified the six hub genes as possible targets: *SRC*, *PIK3R1*, *PIK3CA*, *EGFR*, *PTPN11*, and *AKT1*.

“Combined Application of Fluorescence spectroscopy and Principal Component Analysis in Characterisation of Selected Herbhoneys” by Banaś and Banaś [11] investigated the use of front-face fluorescence spectroscopy with principal component analysis (PCA) as a tool for the characterization of selected Polish monofloral honeys (raspberry, lemon balm, rose, mint, black current, instant coffee, pine, hawthorn, and nettle). Potential differences between the examined samples and the interactions between the compounds were examined using the obtained synchronous spectra in conjunction with PCA. By using the emission spectra recorded in the range of 305–500 nm after excitation at 290 nm, this spectral range enabled analysis of tryptophan residues in proteins. However, some flavonoids (quercetin, miricetin, kaempferol and chrysin), common in many honey samples, may quench tryptofan fluorescence to various extents. Differences in tryptophan intensity might be related to the total content of flavonoids and the concentration of individual compounds from this group, as well as different fluorescence quenching abilities. Emission spectra recorded in the wavelength range of 380–600 nm (λex 375 nm), made it possible to track differences in the content of other phenolic compounds whose fluorescence could not be accurately observed in the spectral range of 305–500 nm, as well as those compounds whose fluorescence intensity was disturbed by the presence of the tryptophan fluorescence band. It was possible to ascertain the immediate environment of tryptophan in the tested products and infer indirectly the presence of flavonoids like quercetin, myricetin, kaempferol, and chrysin that quench its fluorescence by comparing the fluorescence intensity of this amino acid with the location of the band maximum.

“The Comparison of Honey Enriched with Laboratory Fermented Pollen vs. Natural Bee Bread in Terms of Nutritional and Antioxidant Properties, Protein In Vitro Bioaccessibility, and Its Genoprotective Effect in Yeast Cells” by Miłek et al. [12] investigated the nutritional value and bioactivity of honey enriched with a 10% addition of natural bee bread and its substitutes obtained as a result of laboratory fermentation of bee pollen. Using an in vitro static digestion model, the bioaccessibility of proteins, physicochemical parameters, and antioxidant characteristics were examined. The yeast model was used to investigate the bioactivity of the enriched honeys that were produced. The protein from the evaluated products showed good bioaccessibility during in vitro digestion. Using a qualitative spot test, the products’ capacity to protect yeast cells from oxidative stress caused by hydrogen superoxide was shown to be stronger in the case of enriched honey than in pure rapeseed control honey. Significant inhibition of the growth of both strains of yeast exposed to bee pollen-enriched honeys was also demonstrated. Additionally, all examined samples exhibited strong genoprotective activity against genotoxic effects induced by zeocin, and a decrease in DNA double-strand breaks was noticed by a minimum of 70%. The obtained results confirm the benefits of combining honey with both bee bread and its laboratory-obtained substitute. Although new product proposals appear to be potential dietary supplements, animal feeding trials should be used to confirm their nutritional value as best as possible.

“Phytochemical and Bioactivity Studies on *Hedera helix* L. (Ivy) Flower Pollen and Ivy Bee Pollen” by Sen et al. [13] investigated the chemical profiles by high-performance thin-layer chromatography (HPTLC), contents of phenolic marker compounds and pharmacological activities of *Hedera helix* L. (ivy) bee pollen samples and ivy flower pollen grains from Türkiye and Slovenia. The marker compounds afzelin, platanoside and quercetin-3-*O*-β-glucopyranosyl-(1→2)-β-galactopyranoside were common to both bee pollen and flower pollen. They were isolated from bee pollen, and their structures were elucidated by nuclear magnetic resonance (NMR) and mass spectrometry (MS). These three compounds, as well as chlorogenic acid and 3,5-dicaffeoylquinic acid (found in flower pollen), were also quantified. Quercetin-3-*O*-β-glucopyranosyl-(1→2)-β-galactopyranoside was the most bioactive marker compound since it showed the highest antioxidant activity measured by 2,2-diphenyl-1-picrylhydrazyl (DPPH), cupric reducing antioxidant capacity (CUPRAC), ferric reducing antioxidant power (FRAP) and superoxide dismutase (SOD) activity tests. Moreover, it showed the highest xanthine oxidase (XO) inhibitory activity. HPTLC-bioautography (HPTLC-DPPH and HPTLC-XO) also confirmed its contribution to the bioactivity of the extract. The findings of this study can be applied to apitherapy and manufacturing of bee pollen-based food supplements.

“The Influence of the Chemical Composition of Beeswax Foundation Sheets on Their Acceptability by the Bee’s Colony” by Ledjanac et al. [14] investigated the quality of beeswax foundation from six major producers in Vojvodina, Serbia, by applying the classic analytical procedure for the determination of selected physicochemical parameters and instrumental GC–MS and Fourier transform infrared attenuated total reflection (FTIR–ATR) spectroscopy techniques. FTIR–ATR detected possible paraffin and beef tallow in 72 foundation sheet samples. Any paraffin addition represents adulteration, resulting in a high degree of contamination. During the preparation of re-used beeswax, losses during the process may instigate producers to add cheaper, wax-like substances like paraffin and tallow. FTIR–ATR was complemented with GC–MS. According to this analysis, the paraffin level varied from 19.75 to 85.68%, and no tallow was found during the two-year period. Each manufacturer provided two sheets, which were then put in beehives in wired Langstroth-Ruth frames. The built-cell-based construction was observed every 24 h. Evaluating newly inserted sheets proved that without quality nectar, there is no intensive building, regardless of adulteration. Beeswax is one of the most important products for the well-being of bee colonies.

## 3. Conclusions

“Biological Activities and Chemical Composition of Bee Products and Derivatives” highlights the bioactive potential of compounds derived from bees, particularly propolis and geopropolis, honey and stingless bee honey, bee wax, and bee pollen. Numerous biological activities are shown in the investigations, such as genoprotective, anti-inflammatory, wound-healing, anticancer, antibacterial, anti-glycation, and antioxidant properties. Flavonoids, phenolic compounds, terpenoids, and triterpenes form the majority of the complex and varied chemical compositions of bee products, which are intimately linked to these effects. The chemical profile and biological activity of bee products are significantly influenced by geographic location, botanical source, and environmental conditions. FTIR-ATR, UPLC-QTOF HRMS/MS, NMR, GC-MS, HPTLC, and other advanced analytical methods were essential for determining active molecules and clarifying structure–activity correlations. In order to guarantee the effectiveness and safety of bee products, the presence of adulterants in beeswax foundation sheets and the assessment of propolis extracts were evaluated. The predicted safety of these natural products must be ensured by adhering to other safety regulations, such as the presence of bacterial spores, antibiotics, and pesticide residues in other bee products.

In terms of future prospects, several topics can be researched. For instance, studies on fermented pollen-enriched honey or bee bread offer potential for the creation of functional foods, creating new markets for natural, health-promoting dietary supplements. Following this line of reasoning, other innovative uses may be created. For example, combining bee products with other synthetic or natural substances may improve their efficacy or open up new applications in metabolic disorders, dermatology, or oncology. Understanding the mechanistic studies underlying the biological properties indicated for bee products is important since it may allow for focused therapeutic applications. Examples of previously described molecular pathways impacted by bee-derived products include Nrf2/HO-1 and p38/p53 signaling. Because bee products are as complex and chemically varied as nearly every other natural product, standardization of extraction techniques, concentrations, and formulations is required to allow for repeatability and comparison across studies. The usage of bee products must also be validated by clinical trials in order to transform preclinical findings into evidence-based medical applications. It is necessary to investigate, in an ethical and sustainable manner, how environmental factors and various beekeeping techniques affect the chemical composition and bioactivity of bee products. All of these studies are just a few of the areas that could be explored further in the future to meet long-term objectives for the use of bee products across a range of sectors.

## Data Availability

No new data were created or analyzed in this study.
